# Developing of a Mathematical Model to Perform Measurements of Axial Vertebral Rotation on Computer-Aided and Automated Diagnosis Systems, Using Raimondi's Method

**DOI:** 10.1155/2021/5523775

**Published:** 2021-02-05

**Authors:** José Hurtado-Aviles, Joaquín Roca-González, Konstantsin Sergeevich Kurochka, Jose Manuel Sanz-Mengibar, Fernando Santonja-Medina

**Affiliations:** ^1^Faculty of Medicine, University of Murcia, Murcia, Spain; ^2^Technical University of Cartagena (UPCT), Technical School of Industrial Engineering, Cartagena, Spain; ^3^Director of the Industrial and Medical Electronics Section of the DINTEL Research Group, Cartagena, Spain; ^4^Head of Department of Information Technologies, P.O. Sukhoi State Technical University of Gomel, Gomel, Belarus; ^5^V. de la Arrixaca University Hospital, Department of Traumatology, Murcia, Spain

## Abstract

**Introduction:**

Axial vertebral rotation (AVR) is a basic parameter in the study of idiopathic scoliosis and on physical two-dimensional images. Raimondi's tables are the most used method in the quantification of AVR. The development of computing technologies has enabled the creation of computer-aided or automated diagnosis systems (CADx) with which measurement on medical images can be carried out more quickly, simply, and with less intra and interobserver variabilities than manual methods. Although there are several publications dealing with the measurement of AVR in CADx systems, none of them provides information on the equation or algorithm used for the measurement applying Raimondi's method. *Goal*. The aim of this work is to perform a mathematical modelling of the data contained in Raimondi's tables that enable the Raimondi method to be used in digital medical images more precisely and in a more exact manner.

**Methods:**

Data from Raimondi's tables were tabulated on a first step. After this, each column of Raimondi's tables containing values corresponding to vertebral body width (*D*) were adjusted to a curve determined by AVR = *f* (*d*). Third, representative values of each rotation divided by *D* were obtained through the equation of each column *D*. In a fourth step, a regression line was fitted to the data in each row, and from its equation, the mean value of the *D*/*d* distribution is calculated (value corresponding to the central column, *D* = 45). Finally, a curve was adjusted to the obtained data using the least squares method. *Summary and Conclusion*. Our mathematical equation allows the Raimondi method to be used in digital images of any format in a more accurate and simplified approach. This equation can be easily and freely implemented in any CADx system to quantify AVR, providing a more precise use of Raimondi's method, as well as being used in traditional manual measurement as it is performed with Raimondi tables.

## 1. Introduction

Axial vertebral rotation (AVR) is a basic parameter in the study of idiopathic scoliosis in adolescents [1, 2]. Its measurement is necessary to evaluate the severity of scoliosis and quantify the risk of progression [1, 3–7] for the selection of treatment [3, 4, 8, 9] and for the analysis of orthopaedic surgery [1, 6–8, 10, 11].

Two-dimensional medical images, especially anterior-posterior and lateral X-rays of the full spine when standing [12–14], continue to be the method of choice for diagnosing and monitoring scoliosis [15].

On physical two-dimensional images, Raimondi's tables [16–18] are the most used method in the quantification of AVR [19–21]. This method possesses a more than acceptable degree of reproducibility between observers, and it is easy to apply [19, 21, 22].

**Figure 1:**
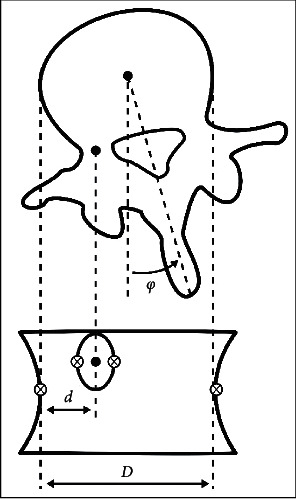
A schematic description of the anatomical references used to measure AVR in two-dimensional images.

Figure 1 shows a schematic description of the anatomical references used to measure AVR in two-dimensional images.

Based on the position of the two closest lateral faces vertebral body points and the two opposite sides of the shadow of the pedicle turned towards the centre of the vertebra in the anteroposterior projection, the software calculates the width of the vertebral body (*D*) and the distance from the centre of the pedicle to the side of the vertebral body (*d*). Adapted by permission from Nature/Springer, European Spine Journal, Vrtovec T, Pernus F, and Likar B, a review of methods was conducted for quantitative evaluation of axial vertebral rotation [7].

The development of computing technologies has enabled the creation of computer-aided or automated diagnosis systems (CADx) with which manual measurement methods on medical images (for instance, Cobb angle, distances, and wedgings) can be applied more quickly, simply, and with less intra and interobserver variabilities [23–28].

The digital X-rays require a software for the clinicians to state a precise diagnosis and treatment, as well as quantify the follow-up changes. These kinds of software are usually not designed to quantify specific AIS parameters, or they are not easily accessible due to their complex use or higher cost.

Although there are several publications dealing with the measurement of AVR in CADx systems, none of them provides information on the equation or algorithm used for the measurement applying Raimondi's method [3, 28–30].

The aim of this work is to perform a mathematical modelling based on the data contained in Raimondi's tables that enables them to be applied on CADx systems, allowing the Raimondi method to be used in digital medical images more precisely and in a more exact manner.

Data from Raimondi's tables were tabulated on a first step. After this, each column of Raimondi's tables containing values corresponding to vertebral body width (*D*) were adjusted to a curve determined by AVR = *f* (*d*). In this mathematical equation, “*d*” represents each value of the column *D*. Third, representative values of each rotation divided by *D* were obtained through the equation of each column D. A representative value for each AVR was obtained this way. The fourth step was to adjust the data included in each row to a regression line, and this equation was used to calculate the average value of the distribution *D*/*d* (middle column value = 45). Finally, a curve was adjusted to the obtained data using the least squares method.

The novelties of our work are (a) to provide an adimentional equation to describe in a theoretically way the empirical data tabulated in Raimondi's tables with more accuracy and less precision error; (b) our mathematical equation allows the Raimondi method to be used in CADx to quantify AVR in medical images without the need for a scale; and (c) this equation can be easily and freely implemented in any CADx system, providing a more precise use of Raimondi's method than on the traditional printed X-ray.

## 2. Materials and Methods

In Raimondi's tables, the data pertaining to one same column correspond to a particular vertebral diameter, *D*, between 20 mm and 70 mm (Table 1). This value is given by the width of the vertebral body *D* projected on the X-ray image.

Within each column, the data correspond to the distance, *d*, between the centre of the shadow of the pedicle furthest from the edge of the vertebra and said edge, in millimetres.

Each pair of values (*D*, *d*) corresponds to a value of AVR between 2° and 60°.

From the distribution of empirical data in Raimondi's tables, a mathematical model that describes the information contained in them in an ideal manner is sought.

First, the data in each column are analysed separately. Using the least squares method, the equation of a curve,

(1) $$\begin{array}{c} f\left({d|}_{D=x}\right)=\text{AVR,} \end{array}$$

is pursued, such that every (*d*, AVR) pair is as close as possible to this curve.

Using the “Statistix 10” software by Analytical Software, a rational function in the form

(2) $$\begin{array}{c} y=\frac{a+bx}{1+cx+dx^{2}}, \end{array}$$

where *a*, *b*, and *c* are the constants, was chosen as the best option.

By adjusting the function model to each set of values in each column of Raimondi's tables, the adjustment values “pseudo *R*^2^” reflected in Table 2 are obtained.

**Figure 2:**
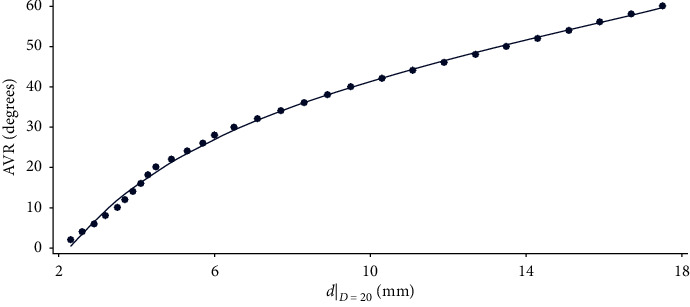
Adjustment of the data in column *D* = 20 of Raimondi's tables to the curve.

The quality of the adjustment of each curve improves as the width of the vertebra, *D*, increases, reaching a maximum of *R*^2^ = 0.9997. The explanation for this may lie in the difficulty of performing a good measurement when this refers to very small distances (Figure 2).

From the value *D* = 48, *R*^2^ decreases slightly until at *D* = 68, we have *R*^2^ = 0.9993.

**Figure 3:**
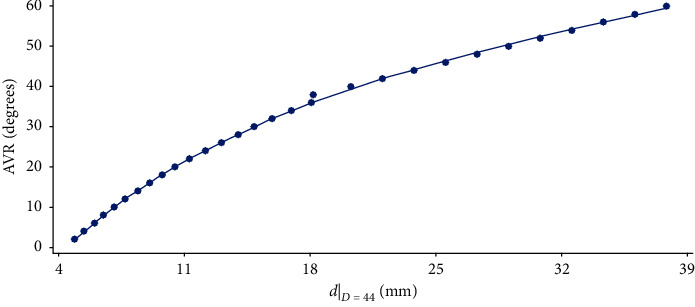
Adjustment of the data in column *D* = 44 of Raimondi's tables to the curve.

It can also be discerned that for *D* = 44, a value of *R*^2^ outside of the collection of values described exists due to an atypical datum (Figure 3).

**Figure 4:**
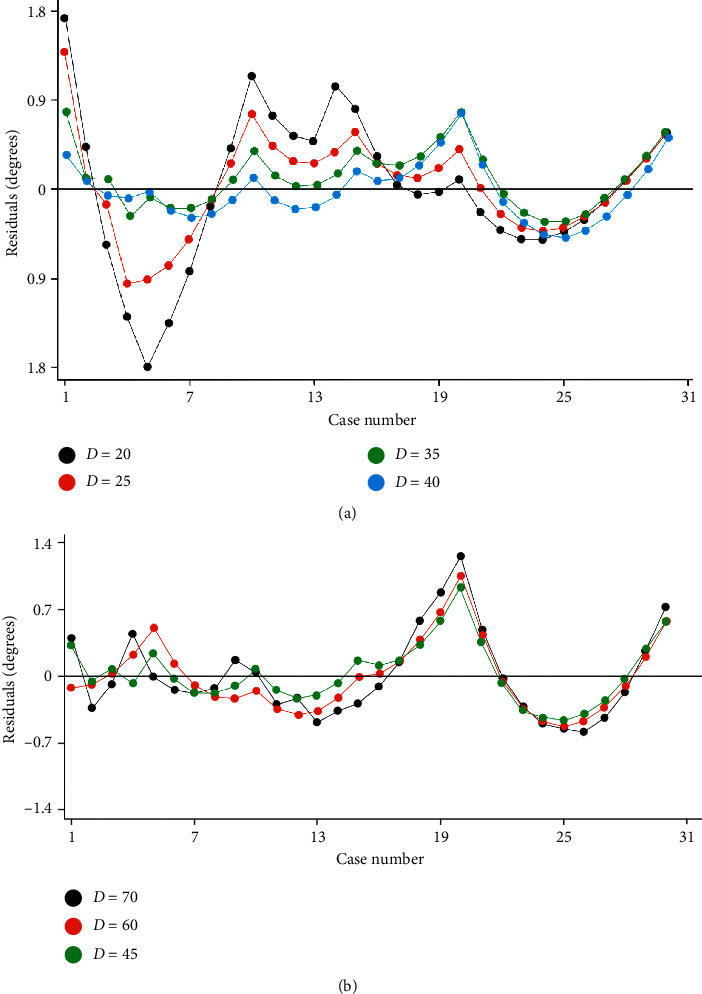
Residuals of the data of various columns from Raimondi's tables regarding their respective regression curves.

Residuals of the data of various columns from Raimondi's tables regarding their respective regression curves can be observed in Figures 4(a) and 4(b).

When using the Raimondi's tables on CADx systems, it is necessary for the vertebrae shown in the digital image to be of sufficient size (20 ≤ *D* ≤ 70) in order to be able to apply the equation [31]. In addition, the dimensions of variables *D* and *d* are given in millimetres.

All pairs of (*D*, *d*) values corresponding to a certain AVR are proportional, meaning they should produce ideally equal *D*/*d* values.

To avoid having to know the scale of the image to measure AVR, the independent variable will have the form *D*/*d* in the calculated equation, such that the value is adimensional.

To calculate the equation

(3) $$\begin{array}{c} f\left(D,d\right)=\text{AVR}, \end{array}$$

the data in Raimondi's tables is replaced by other data obtained in the following manner (Table 3):

- For the set of data in each column, the nonlinear regression curve (equation (2)) is obtained
- From the equation of each regression curve, the *d* values corresponding to the rotations 2°, 4°, 6°,…, 60° are obtained to five decimal places
- The value *D*/*d* is entered to five decimal places in each cell in the tables

In Table 3, the values of each row are very similar. To find the representative value of each rotation, a regression line is adjusted to the data in each row, and from its equation, the average *D*/*d* value of distribution is calculated (value corresponding to the central column, *D* = 45).

**Figure 5:**
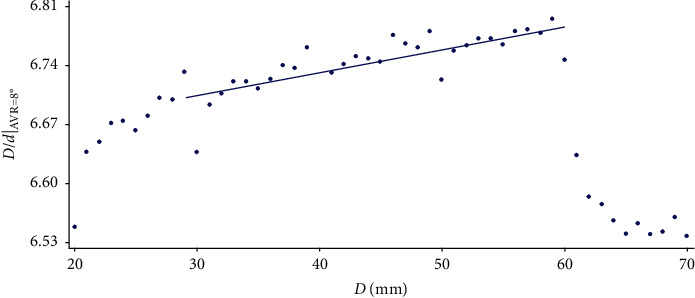
Variation of the *D*/*d* value corresponding to the AVR of 8° as *D* varies.

**Figure 6:**
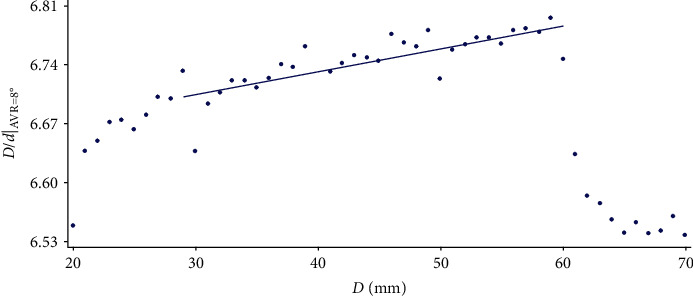
Variation of the *D*/*d* value corresponding to the AVR of 12° as *D* varies.

**Figure 7:**
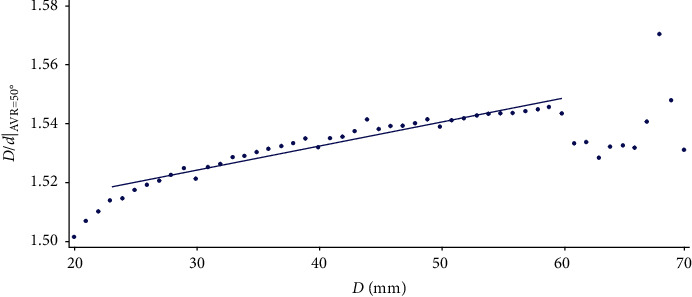
Variation of the *D*/*d* value corresponding to the AVR of 50° as *D* varies.

Figures 5–7 show that as the width of the vertebra increases, the linearity of the point cloud increases. It can also be observed how the general aspect of the data is quite regular, with the exception of those corresponding to *D* = 60 and *D* = 70, where the pattern followed loses its regularity. Consequently, each regression line is calculated by omitting some of the values as shown in the figures.

Table 4 shows the representative *D*/*d* values of each AVR calculated.

## 3. Results

The least squares method is used to adjust a curve to the data of Table 4. Using the “Statistix 10” software, a rational function in the form

(4) $$\begin{array}{c} y=\frac{a+bx+cx^{2}}{1+dx+ex^{2}}, \end{array}$$

where *a*, *b*, *c*, *d*, and *e* are the constants, is chosen. The adjustment obtained with this model is *R*^2^ = 1.

The equation obtained is

(5) $$\begin{array}{c} \text{AVR}=\frac{20.22483-330.5077\left(D/d\right)+33.46082{\left(D/d\right)}^{2}}{1-3.93825\left(D/d\right)-1.322272{\left(D/d\right)}^{2}}. \end{array}$$

Convergence criterion met after 16 iterations.

  Residual SS (SSE): 9.722*E* − 04
  Residual MS (MSE): 3.889*E* − 05
  Standard deviation: 6.236*E* − 03
  Degrees of freedom: 25
  AICc: −294.46
  Pseudo *R*^2^: 10000

## 4. Discussion

AVR is a necessary parameter in being able to correctly evaluate and provide a prognosis for monitor and treat scoliosis [1–7].

Raimondi's tables are widely used on physical X-rays due to its notable reliability and ease of use in quantifying AVR [21, 32].

We are not aware that until now, a method to calculate ARV by CADx systems has been developed using the Raimondi method.

The data contained in the Raimondi's tables were obtained experimentally from thousands of measurements on educational and anatomical spines [32]. In consequence, Raimondi recognises that the measurements obtained with his method involve systematic errors [19, 32]. These errors are small in comparison with the real value of the measures made, meaning their presence will not vary the decision on the evaluation or treatment of the patient. However, these errors may propagate along with random errors due to a multitude of variables present in the measuring process such as (A) the loss of information when showing a three-dimensional structure on a flat image; (B) the characteristics of the medical image (e.g., existence of noise that hinders the legibility of the image); (C) limitations of the observer (e.g., exactitude in the identification of measurement references points, need for training, and experience); (D) the particular morphological characteristics of the vertebrae (e.g., deformations, the variability of the interpedicular distance between vertebrae); and (E) the position of the patient in the image.

On the other hand, medical images today reach the medical professional on a CD, and Raimondi's tables cannot be applied to these digital images, when they do not contain information about the scale (that is, the image does not have the scale imprinted on it or the DICOM archive entry does not have this information).

The obtained equation allows the AVR measurement to be implemented in CADx systems with the Raimondi method, allowing more accurate and precise results to be obtained, due to the elimination of the systematic error contained in the Raimondi tables and the use of auxiliary tools that usually incorporate CADx systems, such as enlargement of regions of interest, application of filters, and automatic detection algorithms.

We have not found any previous work that offers an equation or an analogous algorithm to use Raimondi's method in CADx systems that show it openly. The obtained equation is available to be used freely.

The form of the equation obtained also has the advantage that allows the Raimondi method to be used in digital medical images that do not contain information about its scale (for example, images in TIFF format, in DICOM files that do not contain this information or when it is not printed on the same image).

The limitations of our study are that the programming code to implement our equation in any software is not provided. Our equation was tested using the C++ programming language and OpenCV library, so we can recommend these tools to implement the obtained equation. Also, our equation does not innovate in Raimondi's procedure but achieves a linear theoretical model based on its empirical data, improving its validity and reliability. Therefore, the highlight of our study is that this equation can be easily and freely implemented in any CADx system, allowing the use of the Raimondi method in digital medical images, whether or not they contain the scale, in a more exact and precise way.

## 5. Summary and Conclusion

A mathematical equation has been calculated that can be implemented in CADx systems from the mathematical modelling of the empirical data tabulated in Raimondi's tables.

This allows the Raimondi method to be used in digital images of any format more accurately, simply, and quickly, by eliminating the small systematic error existing in the discrete values of the tables and being able to use in conjunction with auxiliary tools in the CADx system such as zooming over regions of interest, algorithms for enhancing medical image structures, adjusting brightness, and contrast.

The equation can be easily and freely implemented in any CADx system.

## Figures and Tables

**Table 1:** Simplified Raimondi's tables with data to obtain AVR. Modification of Raimondi. The quantification of the vertebral rotation in the scoliosis. Comparing experiences [32].

2.3	2.4	2.5	2.6	2.7	…	7.8	AVR = 2°
2.6	2.7	2.8	2.9	3	…	9	AVR = 4°
2.9	3	3.1	3.2	3.4	…	9.8	AVR = 6°
3.2	3.3	3.4	3.6	3.7	…	10.5	AVR = 8°
3.5	3.6	3.8	3.9	4.1	…	11.7	AVR = 10°
3.7	3.8	4	4.2	4.3	…	12.8	AVR = 12°
3.9	4	4.2	4.4	4.6	…	13.9	AVR = 14°
4.1	4.2	4.5	4.7	4.9	…	15	AVR = 16°
4.3	4.5	4.7	4.9	5.2	…	16	AVR = 18°
4.5	4.7	5	5.2	5.5	…	17.3	AVR = 20°
4.9	5.1	5.4	5.7	5.9	…	18.8	AVR = 22°
5.3	5.5	5.8	6.1	6.4	…	20.1	AVR = 24°
5.7	6	6.3	6.6	6.9	…	21.7	AVR = 26°
6	6.4	6.7	7	7.4	…	23.1	AVR = 28°
…	…	…	…	…	…	…	…
17.5	18.3	19.2	20	20.9	…	61.5	AVR = 60°
*D* = 20	*D* = 21	*D* = 22	*D* = 23	*D* = 24	…	*D* = 70	

**Table 2:** Adjustment of data in Raimondi's tables to the function model.

*D*	*R* ^2^
20	0.9981
21	0.9987
22	0.9988
23	0.9989
24	0.9991
25	0.9992
26	0.9992
27	0.9994
28	0.9994
29	0.9994
30	0.9996
31	0.9996
32	0.9997
33	0.9996
34	0.9997
35	0.9997
36	0.9997
37	0.9997
38	0.9997
39	0.9997
40	0.9997
41	0.9997
42	0.9997
43	0.9997
44	0.9993
45	0.9997
46	0.9996
47	0.9997
48	0.9997
49	0.9996
50	0.9996
51	0.9996
52	0.9996
53	0.9996
54	0.9996
55	0.9996
56	0.9996
57	0.9996
58	0.9996
59	0.9995
60	0.9995
61	0.9994
62	0.9994
63	0.9994
64	0.9994
65	0.9994
66	0.9994
67	0.9994
68	0.9993
69	0.9993
70	0.9993

**Table 3:** Raimondi's tables completed with the results of dividing the *D* value of each column by the values obtained on the regression curves.

8.15408	8.27012	8.36228	8.42931	8.46827	…	8.79462	AVR = 2°
7.57368	7.68005	7.73791	7.78601	7.80927	…	7.89888	AVR = 4°
7.04025	7.13739	7.16903	7.20268	7.21409	…	7.16873	AVR = 6°
6.54855	6.637	6.64894	6.67153	6.67412	…	6.537	AVR = 8°
6.09427	6.17446	6.17198	6.18614	6.18232	…	5.98232	AVR = 10°
5.67363	5.74601	5.73329	5.74124	5.73293	…	5.49782	AVR = 12°
5.2834	5.34847	5.32888	5.33234	5.32102	…	5.06923	AVR = 14°
4.92084	4.97903	4.95525	4.95558	4.94246	…	4.69047	AVR = 16°
4.58353	4.6353	4.60946	4.60776	4.59374	…	4.34998	AVR = 18°
4.26939	4.31517	4.28895	4.28608	4.27189	…	4.04111	AVR = 20°
3.97661	4.01684	3.99152	3.98813	3.97433	…	3.76408	AVR = 22°
3.70362	3.73872	3.71525	3.71187	3.69886	…	3.51061	AVR = 24°
3.44904	3.47945	3.45849	3.4555	3.44357	…	3.2801	AVR = 26°
3.21167	3.23781	3.2198	3.21747	3.20683	…	3.06784	AVR = 28°
…	…	…	…	…	…	…	…
1.1265	1.13327	1.13167	1.13336	1.13382	…	1.11129	AVR = 60°
20/*d*	21/*d*	22/*d*	23/*d*	24/*d*	…	70/*d*	

**Table 4:** Representative *D*/*d* values of each AVR.

AVR (°)	*D*/*d*
2	8.8848
4	8.0729
6	7.3639
8	6.7457
10	6.1983
12	5.7112
14	5.2751
16	4.8817
18	4.5261
20	4.2023
22	3.9068
24	3.6362
26	3.3881
28	3.1603
30	2.9495
32	2.7553
34	2.5753
36	2.4087
38	2.2544
40	2.1112
42	1.9784
44	1.8552
46	1.7409
48	1.6348
50	1.5365
52	1.4452
54	1.3607
56	1.2825
58	1.2102
60	1.1434

## Data Availability

The data used to support the findings of this study are available from the corresponding author upon request.

## Nomenclature

*a*, *b*, *c*, *d*, *e*: Constants
*x*: Variable
*D*: The value given by the width of vertebral body projected on the X-ray image, in millimetres
*d*: The distance between the centre of the shadow of the pedicle furthest from the edge of the vertebra and said edge, in millimetres
*R*^2^: The value of the adjustment obtained with an equation to a set of data.
